# Establishment and Evaluation of Cell Models for Bronchopulmonary Dysplasia: Challenges and Prospects

**DOI:** 10.1111/crj.70118

**Published:** 2025-08-27

**Authors:** Zhenzhuang Zou, Li Fu, Jiaying Liu, Bo Huang

**Affiliations:** ^1^ Department of Pediatrics Shenzhen Futian District Maternity & Child Healthcare Hospital Shenzhen China; ^2^ Department of Pediatrics The Fifth Affiliated Hospital of Zunyi Medical University Zhuhai Guangdong China

**Keywords:** bronchopulmonary dysplasia, drug screening, endothelial cells, in vitro models, lung development, premature infants

## Abstract

Bronchopulmonary dysplasia (BPD) is a chronic lung condition primarily affecting premature infants. It significantly impacts their growth, development, and health. Recently, in vitro models have been created, offering new insights into the causes and potential treatments for BPD. This review summarizes the current methods for creating in vitro models of BPD. It also discusses their evaluation criteria and applications in drug testing and biological research. This paper evaluates the advantages and disadvantages of different models.

## Graphical abstract

Bronchopulmonary dysplasia (BPD) is a chronic lung disease affecting preterm infants, with in vitro models offering insights into its causes and treatments. This review covers methods for creating and evaluating these models, discussing their use in drug testing and biological research, and analyzing the advantages and disadvantages of different models.

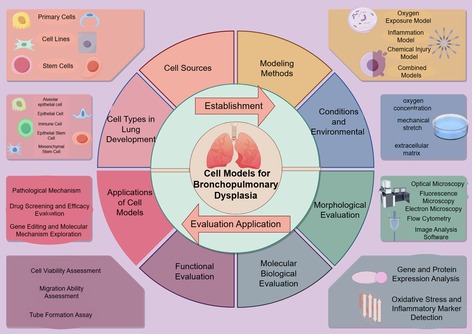

## 1 Introduction

Bronchopulmonary dysplasia (BPD) is a chronic lung disease most commonly observed in preterm infants, characterized by impaired lung development and persistent respiratory issues [1]. Advances in neonatal intensive care have paradoxically contributed to rising BPD prevalence [2]. This condition shift has amplified clinical concerns, as BPD survivors face substantial lifelong morbidity encompassing respiratory and neurodevelopmental complications [3].

The etiology of BPD is multifactorial, involving a complex interplay of genetic predispositions, prenatal and postnatal exposures, and medical interventions [4, 5]. Key factors contributing to BPD include hyperoxic exposures, mechanical ventilation, inflammation, and infection. These factors disrupt normal lung development and cause pathological changes, such as alveolar simplification and pulmonary vascular remodeling [6, 7].

Recently, in vitro models have proven essential for studying the pathophysiology of BPD and for screening potential treatments [8, 9]. These models enable precise control over environmental parameters and genetic factors while permitting mechanistic analysis of cellular responses and molecular pathways driving BPD pathogenesis [10, 11].

The establishment of in vitro models for BPD involves the use of various cell types, including primary cells, cell lines, and stem cells [12, 13]. Primary cells such as human lung fibroblasts, alveolar epithelial cells, and pulmonary microvascular endothelial cells are often used to closely mimic the in vivo environment [14, 15]. Cell lines like A549 and BEAS‐2B provide a more consistent and reproducible platform for high‐throughput screening [16]. Additionally, stem cells, including human embryonic stem cells (hESCs) and induced pluripotent stem cells (iPSCs), offer the potential to model lung development and disease progression in a more physiologically relevant manner [17].

To simulate BPD conditions in vitro, cells are typically exposed to hyperoxic environments, pro‐inflammatory stimuli, or chemical agents, leading to the induction of oxidative stress and inflammatory cascades [18]. These experimental models are indispensable for elucidating cellular mechanisms of injury response and facilitating the discovery of novel therapeutic targets [19, 20].

Evaluation of these cell models includes assessing morphological changes, gene and protein expression, and functional assays such as cell proliferation, migration, and angiogenesis [18, 21]. These evaluations help determine the relevance and reliability of the models in mimicking BPD pathology [19].

This review aims to provide a comprehensive overview of the current methodologies for establishing and evaluating in vitro cell models of BPD. We will discuss the advantages and limitations of different cell types and modeling approaches and highlight their applications in drug screening and biological research. By analyzing the strengths and weaknesses of existing models, we hope to identify potential areas for improvement and future research directions; ultimately contributing to the advancement of BPD research and therapy.

## 2 Key Cell Types in Lung Development and Their Relevance to BPD

In the context of BPD research, understanding the roles of key cell types in lung development is crucial. These cells are fundamental to normal pulmonary function; however, their intricate interactions and specialized functions can be markedly altered in pathological conditions such as BPD [22]. The principal cell types implicated in lung development and their respective roles are delineated as follows:

**Epithelial cells:** Epithelial cells constitute the primary cellular lining of both the airways and alveoli, fulfilling several critical functions:

**Gas exchange:** Alveolar epithelial cells, encompassing Type I and Type II cells, are integral to the process of gas exchange. Type I cells, with their extensive surface area, facilitate efficient oxygen and carbon dioxide exchange. Conversely, Type II cells secrete surfactant, a substance essential for reducing surface tension and preventing alveolar collapse [23].

**Immune surveillance:** Airway epithelial cells function as the first line of defense against pathogens by secreting mucus and antimicrobial peptides, thereby playing a critical role in the host's innate immune defense [24].

**Stromal cells:** Stromal cells, including fibroblasts and other mesenchymal cell types, play a vital role in supporting and regulating lung development:

**Structural integrity:** These cells produce extracellular matrix (ECM) components, such as collagen and elastin, which provide critical structural support, thereby maintaining the lung's shape and elasticity [25].

**Cellular communication:** Stromal cells interact with epithelial cells through the secretion of cytokines and growth factors, which regulate cell proliferation and differentiation and contribute to the establishment of the lung's architectural framework [26].

**Endothelial cells:** Endothelial cells line the blood vessels and are essential for facilitating gas exchange between the blood and alveolar spaces [27]:

**Blood‐gas barrier formation:** Endothelial cells, in collaboration with alveolar epithelial cells, form the blood‐gas barrier, which is crucial for efficient gas exchange.

**Inflammatory response:** In response to inflammation or injury, endothelial cells rapidly facilitate immune cell migration and the release of inflammatory mediators [28].

**Immune cells:** The pulmonary immune system comprises various cell types, including macrophages, lymphocytes, and dendritic cells, each playing distinct roles:

**Macrophages:** Pulmonary macrophages are instrumental in the phagocytosis of inhaled particles and pathogens, and they modulate local immune responses through the release of cytokines [29, 30, 31, 32].

**Lymphocytes:** T cells and B cells are central to the adaptive immune response within the lung, participating in the recognition and elimination of pathogens [33, 34].

**Stem/progenitor cells:** The lung harbors several stem and progenitor cell populations that can be activated to repair tissue damage:

**Epithelial stem cells:** These cells, typically in a quiescent state, have the capacity to proliferate and differentiate into various epithelial cell types to repair damaged regions [35].

**Mesenchymal stem cells:** These cells can differentiate into fibroblasts and other stromal cell types, contributing to ECM reconstruction and structural support [36].

**Cellular interactions:** The interactions among these diverse cell types are crucial for normal lung development:

**Signaling pathways:** Cellular communication is mediated through growth factors and cytokines, which influence cellular processes such as proliferation, migration, and differentiation. For instance, epithelial cells can modulate stromal cell behavior via transforming growth factor (TGF‐β) [37].

**Direct cellular contact:** Direct interactions between cells affect their physiological states, ensuring proper lung development and function [38].

Understanding these cell types and their functions is essential for elucidating the pathogenesis of BPD and for advancing therapeutic strategies. Ongoing advancements in single‐cell genomics and related technologies promise to further enhance our comprehension of these cells, offering novel insights into lung development and associated pathologies [39].

## 3 Establishment of BPD Cell Models

### 3.1 Cell Sources and Selection Criteria

The establishment of in vitro cell models for BPD research necessitates the careful selection of appropriate cell sources. Primary cells, cell lines, and stem cells each offer distinct advantages and limitations. Primary cells, such as human lung fibroblasts, alveolar epithelial cells, and pulmonary microvascular endothelial cells, closely mimic the in vivo environment and maintain the physiological characteristics of the original tissue. These cells are invaluable for studying the cellular and molecular mechanisms underlying BPD, as they provide a more accurate representation of the disease state [40]. However, primary cells are often limited by their finite lifespan and donor variability, which can introduce potential challenges to study reproducibility [41] (Table 1).

**TABLE 1:** Summary of cell sources for in vitro modeling of bronchopulmonary dysplasia.

Cell source	Description	Advantages	Limitations	Applications in BPD research	References
Primary cells	Directly isolated from human tissues	High physiological relevance; closely mimics in vivo conditions	Finite lifespan; donor variability affects reproducibility	Key for studying cellular and molecular mechanisms of BPD	[22, 23]
Human alveolar epithelial cells (AECs)	Isolated from human lung tissue; differentiates into Type I and II cells	Essential for studying alveolar injury, surfactant production, and gas exchange	Difficult to maintain phenotype in culture; limited in long‐term studies	Alveolar injury and repair, surfactant dynamics	[24, 25, 26, 27, 28]
Human pulmonary microvascular endothelial cells (HPMECs)	Isolated from lung microvasculature	Important for studying vascular aspects, endothelial dysfunction, and angiogenesis	Requires specific culture conditions; challenging to isolate in large quantities	Vascular remodeling, endothelial permeability	[33, 34]
Human bronchial epithelial cells (HBECs)	Obtained from bronchial brushings or biopsies	Easier to obtain; useful for studying airway components	Represents bronchial, not alveolar pathology; may not fully capture BPD	Airway inflammation, mucociliary clearance	[35, 36]
Human lung fibroblasts (HLF)	Isolated from lung tissue	Important for studying fibrotic processes and lung remodeling; easy to culture	May not represent full diversity of cell populations involved in BPD	Lung remodeling, repair, and regeneration	[37]
Cell lines	Cultured indefinitely; genetically stable	Ease of culture; consistent and reproducible results	May not fully replicate in vivo conditions; limited physiological relevance	Suitable for large‐scale experiments and high‐throughput studies	[38, 39, 40, 41, 42, 43, 44]
A549 cells	Derived from human lung carcinoma	Useful for studying alveolar epithelial cell functions and surfactant production	May not replicate complex in vivo interactions	Alveolar injury and surfactant dynamics	[39]
BEAS‐2B cells	Originates from human bronchial epithelium	Investigates bronchial epithelial functions and responses	Immortalized; may not reflect primary cell characteristics	Bronchial epithelial responses and mechanisms	[41, 42, 43, 44]
Human umbilical vein endothelial cells (HUVECs)	Isolated from umbilical vein	Easy to culture; useful for studying endothelial functions	Not lung‐specific; may not fully mimic lung vasculature	Angiogenesis, endothelial response to injury	[ 71 ]
Stem cells	Includes hESCs and iPSCs; can differentiate into various lung cell types	Versatile for modeling lung development; patient‐specific modeling	Complex and time‐consuming differentiation protocols	Advanced modeling, 3D cell models, patient‐specific studies	[45, 46, 47]

#### 3.1.1 Primary Cells

Primary cells are directly isolated from human tissues, making them highly relevant for studying the cellular and molecular mechanisms of BPD. Key primary cells used in BPD research include:

**Human alveolar epithelial cells (AECs):** Typically isolated from human lung tissue, these cells are crucial models for investigating alveolar injury‐repair mechanisms that underlie BPD pathogenesis [42, 43, 44]. AECs possess the capacity to differentiate into Type I and Type II cells, vital for studying surfactant production and gas exchange [45, 46, 47]. However, AECs present significant culture maintenance challenges due to their tendency to lose phenotypic specificity, particularly in long‐term studies. By understanding the impact of culture conditions, cellular senescence, signaling pathways, and employing advanced culture techniques such as three‐dimensional (3D) scaffolds, researchers can better maintain the functional properties of AECs.

**Human pulmonary microvascular endothelial cells (HPMECs):** These cells, isolated from lung microvasculature, are critical for studying the vascular pathology of BPD, including endothelial dysfunction and vascular remodeling [48, 49]. They provide a model for studying angiogenesis and endothelial permeability but require specific culture conditions and are difficult to isolate in large quantities [50, 51, 52].

**Human bronchial epithelial cells (HBECs):** Obtained from bronchial brushings or biopsies, HBECs are used to study the airway‐related mechanisms of BPD, such as mucociliary clearance and inflammatory responses [53]. While easier to obtain than alveolar cells, HBECs primarily represent the bronchial epithelium, which may not fully capture alveolar‐specific pathologies [54]. Therefore, a multifaceted approach that integrates both bronchial and alveolar models is advocated to achieve a more comprehensive understanding of respiratory diseases such as BPD, ensuring that the distinct characteristics of both airway and alveolar compartments are adequately addressed in research.

**Human lung fibroblasts (HLF):** HLFs possess various biological characteristics and functions in the physiological and pathological processes of the lung. They play a crucial role not only in normal lung development and maintenance but also in post‐injury repair and regeneration. However, in diseases like BPD, the function of HLFs may be affected, leading to developmental and repair obstacles. Therefore, in‐depth research on the biological characteristics of HLFs and their role in BPD could provide significant scientific evidence for developing new therapeutic strategies. By understanding the various functions of HLFs and their role in BPD, future research can focus on regulating these cells' functions to improve lung health in premature infants with BPD.

These cells, derived from lung tissue, are important for studying the fibrotic processes in BPD, such as lung remodeling [55]. They are relatively easy to culture and expand, making them suitable for large‐scale experiments, though they may not represent the full diversity of cell populations involved in BPD.

#### 3.1.2 Cell Lines

Cell lines offer several advantages, including ease of culture, unlimited proliferation, and genetic stability, making them popular in BPD research. Commonly used cell lines include:

**A549 cells:** Derived from human lung carcinoma, they have become a prevalent model for studying alveolar epithelial cell functions, particularly in the context of respiratory disease and injury [56, 57]. They are frequently utilized in research to gain insights into surfactant production and the cellular responses to alveolar injury. Despite their utility, it is imperative to recognize the limitations of A549 cells when aiming to replicate the intricate cellular interactions present in primary lung tissues [58].

**Human umbilical vein endothelial cells (HUVECs):** HUVECs have gained prominence as a model for studying endothelial functions, largely due to their accessibility and robust proliferative capacity in vitro. These cells serve as vital tools in researching various vascular processes, including angiogenesis and the endothelial response to injury, which are particularly relevant in conditions such as BPD [59]. Despite their utility, HUVECs are not lung‐specific endothelial cells, which raises questions regarding their relevance in pulmonary studies, particularly in mimicking the unique environment of lung vasculature. Despite their convenience, these cell lines may not fully recapitulate the complex cellular interactions and responses observed in primary cells, potentially limiting their translational relevance [60] (Table 2).

**TABLE 2:** Summary of HUVEC applications in BPD research.

Authors	Publication year	Research focus	HUVECs treatment conditions	Key findings	References
Zhang, Y. et al.	2017	Sex‐specific effects of oxygen toxicity in HUVECs	Room air vs. Hyperoxia (95% O2, 5% CO2)	Male HUVECs showed decreased survival, increased oxidative stress, and impaired angiogenesis under hyperoxic conditions.	[ 71 ]
Zhong, X. et al.	2023	Protective effects of umbilical cord blood‐derived exosomes	Normoxia vs. Hyperoxia (75% O2)	UCB‐EXOs alleviated lung injuries in hyperoxia‐insulted mice by elevating miR‐185‐5p levels.	[ 72 ]
Kim, Y. et al.	2019	Therapeutic potential of WKYMVm hexapeptide in BPD	Normoxia vs. Hyperoxia (80% O2)	WKYMVm attenuated hyperoxia‐induced lung inflammation and injuries by upregulating FPR2 and p‐ERK.	[ 73 ]
Xiang, X. et al.	2020	Metformin's effect on pulmonary vascular development in BPD	Room air vs. Hyperoxia (85% O2)	Metformin enhanced lung vascular development and upregulated Gli1 expression in hyperoxic neonatal mice.	[ 4 ]
Liang, Z. et al.	2023	Protectin DX's role in alleviating hyperoxia‐induced lung injury	Normoxia vs. Hyperoxia (60%–70% O2)	PDX treatment relieved hyperoxia‐induced alveolar simplification and lung inflammation by protecting the pulmonary endothelial glycocalyx via the SIRT1/NF‐κB/HPA pathway.	[ 72 ]
Wang, Y. et al.	2022	Expression profiles of circRNAs, lncRNAs, and mRNAs in UCB exosomes from preterm newborns with BPD	LPS‐induced BEAS‐2B cells and HUVECs	A total of 317 circRNAs, 104 lncRNAs, and 135 mRNAs showed significant differential expression in UCB‐derived exosomes of preterm infants with BPD.	[ 74 ]

Abbreviations: BPD, Bronchopulmonary Dysplasia; HUVECs, Human Umbilical Vein Endothelial Cells.

**BEAS‐2B cells:** Originating from human bronchial epithelium, they have gained significance in respiratory research due to their unique characteristics and experimental utility. These immortalized cell lines serve as a model for investigating various bronchial epithelial functions, providing insights into cellular behaviors, responses to stimuli, and mechanisms underlying respiratory diseases [61, 62]. Their utilization reflects a balance between the need for reliable experimental models and the acknowledgment of their limitations compared to primary cell lines [63] (Table 3).

**TABLE 3:** Summary of BEAS‐2B cell applications in BPF research.

Authors	Publication year	Research focus	BEAS‐2B treatment conditions	Key findings	References
Tiwari et al.	2016	Role of GDF15 in hyperoxic lung injury	Normoxia vs. Hyperoxia (95% O2)	GDF15 expression significantly increased under hyperoxia. Knockdown of GDF15 led to decreased cell viability and increased oxidative stress. p53 is crucial for hyperoxia‐induced GDF15 expression.	[ 75 ]
Hu et al.	2022	Vitamin D's role in hyperoxia‐induced BPD	Normoxia vs. Hyperoxia (85% O2)	Vitamin D treatment ameliorated hyperoxia‐induced apoptosis and inflammation by targeting the mitochondrial pathway and MEK1/2‐ERK1/2 signaling pathway in BEAS‐2B cells.	[ 62 ]
Li et al.	2024	TGF‐α/EGFR signaling in LPS‐induced elastin deposition	LPS‐induced BEAS‐2B cells	TGF‐α/EGFR signaling promotes LPS‐induced abnormal elastin deposition and alveolar simplification. EGFR inhibition with erlotinib partially rescued these effects and improved alveolar development.	[ 76 ]
Dinu et al.	2016	Mechanistic role of CYP1B1 in oxygen‐mediated toxicity	Normoxia vs. Hyperoxia (95% O2)	Hyperoxia led to decreased CYP1B1 levels, contributing to cytotoxicity. CYP1B1 overexpression attenuated hyperoxic cytotoxicity, suggesting a role in oxygen toxicity.	[ 77 ]
Meng et al.	2022	lncRNA SNHG6 in hyperoxia‐induced lung cell injury	LPS‐induced BEAS‐2B cells	SNHG6 mediates hyperoxia‐induced lung cell injury via regulating miR‐335 to activate the KLF5/NF‐κB pathway. Knockdown of SNHG6 alleviated hyperoxia‐induced cell injury.	[ 61 ]
Ji et al.	2020	LncRNA CASC2 in neonatal lung injury	Normoxia vs. Hyperoxia (60%–70% O2)	CASC2 targets CAV1 by competitively binding with miR‐194‐5p to inhibit neonatal lung injury. Overexpression of CASC2 alleviated hyperoxia‐induced lung injury by downregulating the TGF‐β1 signaling pathway.	[ 78 ]

Abbreviation: BPD, Bronchopulmonary Dysplasia.

#### 3.1.3 Stem Cells

Stem cells, including hESCs and iPSCs, hold significant potential for advancing BPD research. These cells possess the ability to differentiate into various lung cell types, providing a versatile platform for modeling lung development and disease mechanisms. iPSCs offer the advantage of patient‐specific modeling, allowing for the study of genetic and environmental factors contributing to BPD [64, 65]. The use of stem cells also facilitates the development of 3D cell models and organoids, which can more accurately mimic the architecture and function of the lung tissue [66, 67]. Despite these advances, the differentiation protocols for generating specific lung cell types from stem cells can be complex and time‐consuming, requiring optimization for reproducibility and efficiency [68, 69].

In summary, the choice of cell source for BPD in vitro models depends on the specific research objectives and the balance between physiological relevance and practical considerations. Primary cells offer high fidelity to in vivo conditions, cell lines provide ease of use and consistency, and stem cells offer versatility and the potential for advanced modeling techniques. Each cell type contributes uniquely to the understanding of BPD pathogenesis and the development of therapeutic strategies [70].

Selecting the appropriate cell sources for BPD research requires careful consideration of the specific research objectives. Primary cells provide high physiological relevance, making them ideal for studies focusing on replicating in vivo conditions. Cell lines offer consistency and are well‐suited for large‐scale and high‐throughput studies. Stem cells provide advanced modeling capabilities, including patient‐specific and developmental studies, while rodent models offer valuable insights despite their limitations in human applicability. Balancing these factors is essential for advancing our understanding of BPD pathogenesis and developing effective therapeutic strategies.

### 3.2 Culture Conditions and Environmental Simulation

When establishing in vitro cell models for BPD, it is crucial to simulate the in vivo culture conditions and environment. First, simulating hypoxic and hyperoxic conditions is a key factor in BPD research. Preterm infants are often exposed to a hyperoxic environment, leading to oxidative stress and lung injury. In vitro models can replicate these conditions by adjusting the oxygen levels in the culture medium. For instance, subjecting cells to 21% oxygen (normoxia) and 85% oxygen (hyperoxia) can replicate the cell damage and inflammatory responses associated with BPD [79]. Additionally, hypoxic conditions (e.g., 1%–5% oxygen) are also used to study the mechanisms underlying lung underdevelopment in preterm infants [80, 81]. These studies demonstrate that changes in oxygen concentration significantly affect cell proliferation, differentiation, and function.

Second, mechanical stretch is another critical factor in simulating lung injury mechanism. During mechanical ventilation, the repetitive mechanical stretching of preterm infant lung tissue is a primary contributor to BPD. Mechanical stretch devices in vitro can simulate the impact of this mechanical stress on cells. For example, using the Flexcell system to apply cyclic stretch to cells allows researchers to investigate changes in cell morphology, gene expression, and function [82]. Studies have found that mechanical stretch can induce inflammatory responses and oxidative stress in cells, exacerbating lung injury [83, 84].

Finally, the influence of the ECM cannot be overlooked. The ECM provides physical support for cells and modulates cellular behavior and function by interacting with cell surface receptors. In vitro models can simulate the in vivo microenvironment by using different types of ECM coatings (such as collagen, fibronectin, and laminin) to study the role of the ECM in BPD [85]. For instance, studies have shown that changes in ECM components can affect the proliferation, migration, and differentiation of alveolar epithelial and endothelial cells, thereby influencing lung development and repair [86].

In summary, by recapitulating key pathological factors including hypoxic and hyperoxic conditions, mechanical stretch, and the influence of the ECM, more physiologically relevant BPD models can be established in vitro. These models not only help in understanding the pathogenesis of BPD but also provide valuable tools for drug screening and the development of therapeutic strategies.

### 3.3 Modeling Methods

#### 3.3.1 Oxygen Exposure Model

The oxygen exposure model serves as a prevalent technique for simulating the hyperoxic environments frequently encountered by preterm infants, which can precipitate BPD. This methodology entails subjecting cultured cells to elevated oxygen concentrations, typically between 60% and 95%, thereby replicating the oxidative stress conditions prevalent in neonatal intensive care settings. Research has indicated that hyperoxia significantly induces oxidative stress and inflammation, culminating in cellular damage and hindered lung development—key characteristics associated with BPD [71, 86]. For example, in vitro investigations utilizing alveolar epithelial and endothelial cells have revealed that hyperoxic exposure leads to heightened production of reactive oxygen species (ROS), mitochondrial dysfunction, and the activation of pro‐inflammatory signaling pathways [87, 88]. These cellular alterations closely mirror the pathological changes observed in the lungs of infants diagnosed with BPD, thereby establishing the oxygen exposure model as an essential instrument for probing the disease's underlying mechanisms and exploring potential therapeutic strategies. These cellular responses closely resemble the pathological changes observed in the lungs of infants with BPD, making the oxygen exposure model a valuable tool for studying the disease's mechanisms and potential therapeutic interventions.

#### 3.3.2 Inflammation Model

The inflammation model represents another vital approach for investigating BPD in vitro. This model typically employs bacterial endotoxins, such as lipopolysaccharide (LPS), or pro‐inflammatory cytokines to elicit an inflammatory response in cultured cells. LPS, a constituent of the outer membrane of Gram‐negative bacteria, is frequently utilized to replicate the sustained inflammatory microenvironment associated with BPD. The interaction of LPS with toll‐like receptor 4 (TLR4) on the cell surface initiates a series of inflammatory signaling cascades, notably the nuclear factor‐kappa B (NF‐κB) pathway [89, 90]. This activation triggers the release of pro‐inflammatory cytokines, including TNF‐α, IL‐1β, and IL‐6, which contribute to lung injury and disrupted alveolar development [91, 92]. The inflammation model is particularly instrumental in elucidating the role of inflammation in the pathogenesis of BPD and in evaluating anti‐inflammatory therapeutic interventions.

#### 3.3.3 Chemical Injury Model

The chemical injury model utilizes chemical agents, such as bleomycin, to induce lung injury and fibrosis, both of which are hallmark features of BPD. Bleomycin, a chemotherapeutic agent recognized for its capacity to inflict DNA damage and induce oxidative stress, leads to apoptosis and fibrosis within lung tissues [93]. In vitro studies involving lung epithelial cells and fibroblasts have demonstrated that exposure to bleomycin results in increased ROS production, activation of fibrotic pathways, and upregulation of fibrotic markers, including collagen and α‐smooth muscle actin (α‐SMA) [94]. This model is instrumental in clarifying the mechanisms that underlie lung fibrosis in BPD and in assessing potential antifibrotic therapies [95].

#### 3.3.4 Combined Models

Combined models that incorporate multiple interacting factors, such as hyperoxia and inflammation, offer a more holistic representation of the intricate pathophysiological conditions associated with BPD. For instance, subjecting cells to both elevated oxygen levels and LPS can replicate the synergistic effects of oxidative stress and inflammation, commonly observed in preterm infants afflicted with BPD [72, 96, 97]. These integrated models are particularly advantageous for investigating the interactions among different pathogenic factors and for identifying multifaceted therapeutic approaches. Studies have demonstrated that such models can more accurately reflect the cellular and molecular alterations characteristic of BPD, including increased ROS production, the release of inflammatory cytokines, and cellular apoptosis [98, 99].

## 4 Evaluation Systems for Cell Models

### 4.1 Morphological Evaluation

Morphological evaluation serves as a cornerstone for validating in vitro cell models of BPD. This assessment allows for a comprehensive understanding of the cellular alterations that occur under pathological conditions [4]. Key techniques include:

1. Optical microscopy: Fundamental for initial assessments, optical microscopy, combined with staining methods like hematoxylin and eosin (H&E), enables the visualization of cell shape, size, and arrangement. This technique is essential for identifying basic structural changes associated with BPD.
2. Fluorescence microscopy: By utilizing fluorescently labeled antibodies, fluorescence microscopy facilitates the study of specific protein distribution within cells. This method is particularly useful for examining the expression and localization of markers in alveolar epithelial and endothelial cells under BPD conditions.
3. Electron microscopy: Offering superior resolution, electron microscopy (both transmission and scanning) provides insights into the ultrastructural changes within cells. This technique is crucial for investigating microvascular damage and morphological alterations in lung tissue, which are characteristic of BPD [100].
4. Flow cytometry: This technique enables the rapid analysis of large cell populations, assessing parameters such as cell size, shape, and surface marker expression. Flow cytometry is invaluable for quantifying the proportion and activity of cell types implicated in BPD.
5. Image analysis software: Advances in technology have led to the widespread use of image analysis software for quantitative assessment of cell morphology. These tools provide precise measurements of cellular dimensions, contributing to a deeper understanding of how experimental conditions affect cell morphology in BPD models.

These methods provide detailed insights into the cellular architecture and can reveal disruptions in cell–cell junctions, cytoskeletal organization, and ECM composition. Such morphological assessments are crucial for validating the relevance of the cell model to human BPD pathology and for identifying potential therapeutic targets.

### 4.2 Molecular Biological Evaluation

1. Gene and protein expression analysis: Techniques such as quantitative PCR (qPCR), RNA sequencing (RNA‐Seq), Western blotting, and enzyme‐linked immunosorbent assay (ELISA) are employed to analyze the expression levels of key genes and proteins. These methods quantify the expression of critical molecules involved in inflammation, oxidative stress, and cell proliferation, which are relevant to BPD pathogenesis.
2. Oxidative stress and inflammatory marker detection: The evaluation includes the measurement of inflammatory cytokines like TNF‐α and IL‐6, as well as oxidative stress markers such as ROS and nitric oxide synthase (NOS). These assessments help in understanding the underlying mechanisms of BPD and in validating the impact of experimental conditions on lung cell function and development [101].

These methods help in quantifying the expression levels of key genes and proteins involved in inflammation, oxidative stress, and cell proliferation. For example, studies have shown that exposure to hyperoxia leads to upregulation of inflammatory cytokines such as TNF‐α and IL‐6, as well as markers of oxidative stress like ROS and NOS. Additionally, the expression of surfactant proteins and markers of alveolar epithelial cell differentiation can be assessed to evaluate the impact of experimental conditions on lung cell function and development. These molecular insights are essential for understanding the pathophysiology of BPD and for developing targeted therapeutic strategies.

### 4.3 Functional Evaluation

Functional evaluation assesses the physiological and biochemical activities of cells in the model, providing a comprehensive understanding of their behavior under pathological conditions. This includes assays for cell viability, proliferation, migration, and differentiation.

1 **Cell viability assessment:** In the study of BPD, evaluating cell viability is a crucial aspect for assessing therapeutic efficacy and drug toxicity. Understanding the methodologies used for cell viability measurement is essential. This section provides a detailed overview of common techniques, including the MTT assay, CCK‐8 assay, and others.

#### 4.3.1 MTT Assay

The MTT assay is a classical method for assessing cell viability based on mitochondrial dehydrogenase activity. This assay involves the reduction of MTT, a yellow tetrazolium salt, into insoluble purple formazan crystals by viable cells. The absorbance is measured at 570 nm, which correlates with the number of viable cells. While the MTT assay is highly sensitive and applicable to various cell types, it is limited by its cytotoxicity and its use as an endpoint measurement, which does not allow for real‐time monitoring of cell viability [102].

#### 4.3.2 CCK‐8 Assay

The CCK‐8 assay is an improved method that utilizes the water‐soluble tetrazolium salt WST‐8. This assay measures cell viability by detecting the reduction of WST‐8 to a water‐soluble formazan dye, with absorbance read at 450 nm. The CCK‐8 assay is less toxic to cells, making it suitable for dynamic and long‐term experiments. However, it is relatively more expensive, and its accuracy can be affected by certain components in the culture medium [103].

In addition to the MTT and CCK‐8 assays, several other cell viability assays are widely used. For instance, the XTT assay is similar to the MTT assay but generates a soluble product, simplifying the procedure [104]. The WST‐1 assay is akin to the CCK‐8 assay and is favored for high‐throughput screening [105]. Moreover, real‐time cell analysis (RTCA) employs electrical impedance to monitor cell viability continuously, offering a dynamic view of cell growth and toxicity responses [106].

In conclusion, selecting the appropriate cell viability assay is critical in BPD research. Researchers should choose the method that best suits their experimental needs and conditions. As technology advances, more noninvasive cell viability assays will emerge, providing more accurate and real‐time monitoring options for BPD studies.

2 **Migration ability assessment:** Wound healing or Transwell migration assays assess the impact of BPD conditions on the migratory capacity of endothelial cells, which is essential for angiogenesis and tissue repair [107]

#### 4.3.3 Scratch Assay

The scratch assay is a well‐established technique for studying cell migration and proliferation. It involves creating a “scratch” in a confluent cell monolayer and monitoring the closure of this gap over time as cells migrate to fill the wound [108]. The scratch assay is easy to perform and allows for real‐time observation of cell movement. However, its accuracy can be compromised by inconsistencies in scratch width; it is primarily qualitative, providing limited quantitative data [109].

#### 4.3.4 Transwell Migration Assay

The Transwell migration assay offers a more quantitative approach by measuring the migration of cells through a porous membrane in response to a chemoattractant [110]. Cells are placed in the upper chamber of a Transwell insert, and their movement towards the chemoattractant in the lower chamber is quantified after a specified incubation period. This assay is highly effective for studying directional cell migration and can simulate aspects of the in vivo environment. However, it may not fully capture the complexity of cell migration as it occurs in living organisms [111, 112, 113].

Beyond the scratch and Transwell assays, other methods are frequently employed to assess cell migration. For example, the Boyden chamber assay, a variant of the Transwell assay, operates on a smaller scale and allows for controlled experimental conditions [114]. Additionally, real‐time imaging techniques enable continuous monitoring of cell migration, while matrix gel migration assays offer insights into how cells navigate through extracellular matrices [115]. Immunofluorescence staining can further elucidate the molecular mechanisms underlying cell migration [116].

In conclusion, the choice of cell migration assay in BPD research should be guided by the specific objectives of the study. Each method has its strengths and limitations, and the selection of the appropriate assay is crucial for obtaining accurate and meaningful data. As research in this area progresses, new methods will likely emerge, offering more sophisticated tools for investigating cell migration in BPD.

3 **Tube formation assay:** The tube formation assay is a crucial in vitro technique in BPD research, used to evaluate angiogenesis by assessing the ability of endothelial cells to form capillary‐like structures on a basement membrane matrix [117]. This process simulates blood vessel formation and is vital for understanding the impact of various therapeutic interventions on vascular development. BPD is characterized by impaired angiogenesis due to incomplete lung development and oxidative stress from oxygen therapy [118]. Enhancing angiogenesis to improve oxygen delivery is a central therapeutic objective, and the tube formation assay helps evaluate how different strategies, such as pharmacological agents or cell‐based therapies, can restore vascular integrity in BPD.

The assay's high‐throughput capabilities make it an invaluable tool for screening numerous compounds to identify those with significant angiogenic potential. It has been instrumental in demonstrating the pro‐angiogenic effects of exosomes from maternal or stem cells, highlighting their therapeutic potential. Moreover, the assay aids in investigating key biomarkers and signaling pathways, such as VEGF and FGF, integral to the angiogenic process. Future research should address challenges like understanding interactions between endothelial cells and surrounding cell types to provide a more comprehensive understanding of angiogenesis. Bridging the gap between in vitro findings and clinical applications remains a critical step in developing effective BPD therapies, potentially through innovative combinatory cell and growth factor therapies. These assays evaluate the angiogenic potential of endothelial cells, a function often impaired in BPD [119].

These functional assays provide critical data on how experimental conditions affect cell behavior and can help in identifying potential therapeutic interventions that can restore normal cell function and promote lung repair [120, 121].

## 5 Applications of Cell Models in BPD Research

### 5.1 Pathological Mechanism Research

Cell models have significantly advanced our understanding of the pathological mechanisms underlying BPD. These models allow researchers to dissect the complex interplay of factors such as oxidative stress, inflammation, and mechanical ventilation that contribute to the disease. For instance, studies using alveolar epithelial cells and lung fibroblasts have elucidated the role of oxidative stress in cellular injury and apoptosis, which are critical events in BPD pathogenesis [122]. Additionally, inflammation‐induced models using cytokines like TNF‐α and IL‐6 have provided insights into the inflammatory pathways that exacerbate lung damage [123, 124]. These experimental models have proven pivotal in elucidating critical molecular mediators, like NF‐κB and TGF‐β, which mediate the inflammatory response and fibrosis in BPD [125, 126]. By replicating the pathological conditions of BPD in vitro, these cell models offer a controlled environment to investigate disease mechanisms and high‐throughput screening of therapeutic interventions.

### 5.2 Role of Vascular Endothelial Cells (VECs) in BPD

VECs play a pivotal role in lung development and angiogenesis, processes that are critically impaired in BPD [127]. In vitro models using VECs have demonstrated how hyperoxia and inflammation disrupt endothelial function, leading to impaired angiogenesis and vascular remodeling [128]. These studies have shown that oxidative stress induces endothelial cell apoptosis and downregulates the expression of angiogenic factors such as VEGF [129]. Furthermore, research has highlighted the potential of endothelial progenitor cells (EPCs) in repairing damaged vasculature, suggesting a therapeutic avenue for BPD [86]. By focusing on the endothelial aspects of BPD, these models provide valuable insights into the vascular component of the disease and open up new possibilities for targeted therapies.

### 5.3 Drug Screening and Efficacy Evaluation

Cell models are indispensable tools for drug screening and evaluating therapeutic efficacy in BPD. These models allow for high‐throughput screening of potential drugs under controlled conditions that mimic the BPD environment. For example, the use of A549 cell lines and primary alveolar epithelial cells has facilitated the screening of antioxidants and anti‐inflammatory agents that could mitigate oxidative and inflammatory damage [130, 131]. Additionally, 3D organoid models have been employed to assess the efficacy of novel therapeutics in a more physiologically relevant context [132, 133]. These models have also been used to evaluate the potential side effects and toxicity of new drugs, ensuring their safety before clinical trials [132, 134, 135]. By providing a platform for rapid and efficient drug testing, cell models accelerate the development of effective treatments for BPD.

### 5.4 Gene Editing and Molecular Mechanism Exploration

Gene editing technologies, such as CRISPR/Cas9, have revolutionized the study of BPD by enabling precise manipulation of genes involved in the disease. In vitro cell models have been used to knock out or overexpress specific genes to study their roles in BPD pathogenesis [136, 137]. For instance, gene editing has been employed to investigate the function of genes related to oxidative stress response, such as Nrf2, and their impact on cell survival and inflammation [138]. These models have also facilitated the study of epigenetic modifications and their contribution to BPD, providing a deeper understanding of the molecular mechanisms driving the disease [139, 140]. By leveraging gene editing tools, researchers can uncover novel molecular targets and pathways, paving the way for innovative therapeutic strategies.

### 5.5 Research Examples

Several research examples highlight the utility of cell models in advancing BPD research. One notable study investigated the role of gasdermin D (GSDMD) in hyperoxia‐induced BPD using gene‐edited alveolar epithelial cells. The study found that GSDMD deficiency attenuated cell death and inflammation, suggesting a potential therapeutic target for BPD [141]. Another example involves the use of stem cells, such as mesenchymal stem cells (MSCs) and iPSCs, to explore their regenerative potential in BPD [142]. These studies have shown that stem cell therapy can enhance lung repair and reduce inflammation, offering promising avenues for treatment. These examples underscore the versatility and effectiveness of cell models in uncovering new insights and developing novel therapies for BPD.

### 5.6 Present Research

Mesenchymal stromal/stem cells (MSCs), as core members of the stem cell family, have become a research focus in exploring lung injury‐repair mechanisms due to their multidirectional differentiation potential (including differentiation into lung cells), immunomodulatory properties, anti‐inflammatory and antioxidant stress capabilities, and tissue regeneration functions. MSCs primarily exert therapeutic effects through paracrine signaling, with extracellular vesicles (EVs) playing a crucial role in this process [11]. Secreted by various cells, EVs regulate recipient cells' immune functions, developmental processes, tissue homeostasis, and disease mechanisms through endocytosis and signal transduction in target cells. Recent studies demonstrate that MSC‐EVs can protect lungs by suppressing inflammatory responses, improving pulmonary function, and alleviating pulmonary hypertension [8, 143, 144]. Notably, emerging research reveals that angiogenesis‐related growth factors (such as VEGF) released by MSCs are predominantly stored within EVs (particularly exosomes) [145, 146], suggesting MSC‐derived exosomes may play a key role in BPD treatment through VEGF‐driven angiogenesis.

In hyperoxia‐induced BPD rat models, intratracheal administration comparisons between MSCs and EVs showed both could mitigate hyperoxic lung injury, with MSC‐EVs demonstrating superior efficacy in promoting alveolar development and pulmonary angiogenesis [108, 147, 148]. Beyond animal models, cellular‐level research has advanced significantly. For instance, studies on placental mesenchymal stem cells (PMSCs) reveal that PMSCs promote trophoblast ATP generation by regulating calcium channel expression and inducing mild oxidative stress, while their derived exosomes significantly alleviate LPS‐induced pulmonary lipid peroxidation and apoptosis. Furthermore, PMSC‐derived exosomes can be internalized by VECs, enhancing angiogenesis‐related gene expression and promoting vascular formation and migration [14, 29, 149]. These findings provide theoretical support for PMSCs in treating BPD‐associated metabolic abnormalities and vascular developmental disorders, though underlying mechanisms require further exploration.

In basic research, primary lung cell co‐culture models (e.g., human alveolar type II cells co‐cultured with fibroblasts/MSCs) are widely used to investigate hyperoxia's impact on lung development. Studies confirm that hyperoxia abnormally activates the Wnt/β‐catenin pathway through the NF‐κB‐Wnt5a signaling axis, leading to alveolarization impairment and fibrosis [150], suggesting Wnt5a as a potential therapeutic target for chronic lung diseases including BPD. Such in vitro models not only simulate pathological responses but also serve as drug screening platforms to reduce animal experimentation. For example, the metabolite itaconic acid (ITA) alleviates hyperoxia‐induced alveolar type II epithelial cell apoptosis by regulating TFEB‐mediated autophagic flux, while autophagy inducers (rapamycin and LiCl) enhance cell survival by restoring autophagic activity [151, 152]. These discoveries provide new directions for developing autophagy‐targeted BPD therapies.

Currently, MSC‐based therapies have demonstrated good safety in preclinical studies [153, 154]; while other cell types (e.g., primary lung epithelial cells and fibroblasts) are more commonly used to decipher BPD's molecular mechanisms. With deepening research, the integration of multilevel evidence (cellular, animal, and clinical) will accelerate the translation of precision treatment strategies for BPD into clinical applications.

## 6 Limitations of Current Research and Future Directions

Research into BPD through cellular models has revealed significant limitations that hinder our understanding and treatment of this complex condition. Current models, often criticized for their simplicity, fail to fully replicate the intricate interactions among cellular and molecular events occurring in vivo. These models frequently overlook essential components of the lung microenvironment, such as the ECM and immune cell interactions, which are critical for studying lung development and the pathophysiological mechanisms underlying BPD [155]. Consequently, findings from these models may lack physiological relevance, limiting their translational potential.

### 6.1 Reproducibility and Reliability Challenges

Another major issue with these models is the lack of reproducibility and reliability of the data they generate [156]. Variability in culture conditions, cell types, and experimental techniques often results in inconsistent findings across different studies, making it difficult to validate results and raising concerns about the reliability of conclusions drawn from these models.

### 6.2 Future Directions: Advancing BPD Models

To overcome these limitations and advance the field, future BPD research should focus on developing more sophisticated models that better mimic lung development and the pathophysiology of BPD [157]. Innovations such as 3D culture systems and organ‐on‐a‐chip technologies offer the potential to create more physiologically relevant environments for studying cellular behaviors and responses within the context of BPD. These advanced models could provide deeper insights into the mechanisms driving BPD, thereby enabling the development of more targeted therapeutic strategies.

### 6.3 High‐Throughput Screening in BPD Research

Integrating high‐throughput screening techniques into BPD research could significantly enhance the efficiency of drug discovery and therapeutic evaluation. By allowing researchers to simultaneously assess a large number of compounds, high‐throughput screening accelerates the identification of potential treatments and interventions for BPD [158].

### 6.4 Integrating In Vitro and In Vivo Approaches

The future of BPD research may benefit from the strategic combination of in vitro cellular models with in vivo animal studies. This integrated approach would enable researchers to validate in vitro findings in living systems, thereby bridging the gap between in vitro and in vivo research. Such a strategy not only provides a more comprehensive understanding of BPD but also helps assess how well treatments identified in vitro translate into physiological benefits in vivo [159].

In summary, the current use of cellular models in BPD research faces significant challenges related to model complexity, physiological relevance, and data reliability. By developing more advanced models, employing high‐throughput screening, and integrating in vitro with in vivo approaches, the field is well positioned to overcome these obstacles and make substantial progress toward more effective treatments for infants with BPD.

## 7 Discussions

The establishment and assessment of in vitro cell models for BPD represent a significant advancement in understanding the pathogenesis and potential treatment strategies for this chronic lung disease, predominantly affecting premature infants. These models provide a controlled environment to mimic the complex interplay of factors contributing to BPD, such as oxidative stress, inflammation, and mechanical ventilation, which are challenging to study in vivo.

From an expert perspective, the development of various cell models, including primary cells, cell lines, and stem cell‐derived 3D organoids, has enriched the toolbox available for BPD research. Primary cells like human lung fibroblasts, alveolar epithelial cells, and microvascular endothelial cells offer closer physiological relevance, while immortalized cell lines such as A549 and BEAS‐2B provide consistency and ease of use. The advent of hESCs and iPSCs has opened new avenues for generating patient‐specific models that could better recapitulate individual variability in BPD pathophysiology.

Evaluating these models through a combination of morphological, molecular, and functional assessments ensures a comprehensive understanding of their validity and applicability. Techniques like qPCR, Western blotting, and ROS detection for oxidative stress, alongside functional assays for cell migration and angiogenesis, are crucial for characterizing the models' response to BPD‐like conditions. These evaluations not only confirm the models' relevance but also help identify potential therapeutic targets.

However, challenges remain in enhancing the physiological relevance and complexity of these models. Current in vitro systems often fall short in fully mimicking the dynamic and multifactorial nature of BPD seen in vivo. Future research should focus on integrating multi‐cellular and tissue‐level interactions, possibly through co‐culture systems or organ‐on‐a‐chip technologies that can better simulate the lung microenvironment. Additionally, the reproducibility and scalability of these models need to be addressed to ensure they can be widely adopted for high‐throughput drug screening and mechanistic studies.

The implications of in vitro BPD models extend beyond basic research. They hold promise for personalized medicine approaches, allowing for the testing of patient‐specific responses to therapies and potentially leading to more targeted and effective treatments. The integration of gene editing technologies, such as CRISPR‐Cas9, with these cell models could further elucidate the genetic underpinnings of BPD and identify novel therapeutic avenues.

In conclusion, the establishment and evaluation of in vitro cell models for BPD are pivotal for advancing our understanding of this complex disease. By continuing to refine these models and addressing their current limitations, we can enhance their utility in both basic research and clinical applications, ultimately paving the way for more effective interventions for BPD. The ongoing development in this field is anticipated to bring significant breakthroughs, improving the prognosis and quality of life for those affected by BPD.

## Author Contributions

**Zhenzhuang Zou:** data curation; formal analysis; investigation; project administration; writing – original draft. **Li Fu:** data curation; formal analysis; project administration; writing – original draft. **Jiaying Liu:** data curation; formal analysis; project administration; writing – original draft; writing – review and editing. **Bo Huang:** data curation; formal analysis; investigation; project administration (lead); writing – original draft; writing – review and editing (lead).

## Ethics Statement

The authors have nothing to report.

## Conflicts of Interest

The authors declare no conflicts of interest.

## Data Availability

All raw data and code are available upon request.
